# Activities, Housing Situation and Other Factors Influencing Psychological Strain Experienced During the First COVID-19 Lockdown in Switzerland

**DOI:** 10.3389/fpsyg.2021.735293

**Published:** 2021-09-28

**Authors:** Ralph Hansmann, Livia Fritz, Anna Pagani, Garance Clément, Claudia R. Binder

**Affiliations:** ^1^Laboratory for Human Environment Relations in Urban Systems (HERUS), Swiss Mobiliar Chair in Urban Ecology and Sustainable Living, École Polytechnique Fédérale de Lausanne (EPFL), Lausanne, Switzerland; ^2^Transdisciplinarity Lab (TdLab), Department of Environmental Systems Science (D-USYS), Swiss Federal Institute of Technology (ETH Zurich), Zurich, Switzerland; ^3^Laboratory of Urban Sociology (LASUR), École Polytechnique Fédérale de Lausanne (EPFL), Lausanne, Switzerland

**Keywords:** COVID-19, lockdown, well-being, social factors, housing, employment status, leisure activities

## Abstract

**Background:** The Coronavirus disease 2019 (COVID-19) crisis and the corresponding first nationwide lockdown from mid-March to 10 May 2020 engendered considerable psychological strain among people in Switzerland. This study analyzes determinants of changes in subjective levels of psychological strain experienced during the lockdown.

**Methods:** An online survey conducted as part of a larger mixed methods study examined the material and emotional aspects of individual reactions to the lockdown from a socio-ecological perspective. Participants (*N* = 5932) were asked about their personal and employment status, housing features, changes in various activities (e.g., physical activity, watching TV, social media use) and aspects of mental distress and well-being.

**Results:** A substantial share of participants reported to feel depressed (33%) and anxious (43%) more often during the COVID-19 lockdown than before, whereas significantly (*p* < 0.001) less persons reported a decrease of these negative feelings (depressed 17%; anxious 14%). Women, single people, students and people who lost their jobs or were temporally unemployed due to the lockdown experienced a particularly strong increase of subjective psychological strain. Important residential factors reducing subjective psychological strain were the general comfort of the housing situation and having a private garden or multiple types of outdoor space. Considering leisure activities, the strongest positive psychological effect resulted from increased physical activities, followed by reading and cooking. However, 45% of the participants reported a decreased frequency of physical activity during the lockdown compared to before, whereas significantly less persons (26%) reported a corresponding increase (*p* < 0.001).

**Conclusion:** Consistent with other studies, the results indicate a substantial reduction of subjective psychological well-being of the population during the first COVID-19 lockdown in Switzerland. The psychological burdens which the participants experienced differ depending on personal characteristics and situational factors. Negative psychological and economic consequences and gender inequalities should accordingly be carefully considered and actively prevented when designing COVID-19 measures. Supportive economic and social, cognitive and behavioral psychological interventions need to be designed and implemented to maintain the well-being of residents during lockdown.

## Introduction

The first COVID-19 lockdown in Switzerland exposed people to novel circumstances, which presented challenging stressors that had severe impacts on psychological balance and well-being (Kuhn et al., 2021; Gubler et al., 2021; Peterson et al., 2021). Societal and economic lockdown measures imposed substantial burdens on people worldwide, resulting in increased anxiety and depressive emotions (Burhamah et al., 2020; Xiong et al., 2020; Mencaccia and Salvi, 2021; Passavanti et al., 2021; Vermote et al., 2021). During pandemic conditions, psychological strain can be caused by social isolation due to contact restrictions, quarantine measures, school and company closures, and related negative career impacts such as becoming unemployed (Blustein and Guarino, 2020; Prati, 2020; Serafini et al., 2020). Corresponding experiences and fears of illness, social isolation, and economic and social decline put severe psychologically pressure on people and substantially reduced their happiness and well-being (Daly et al., 2020; Dale et al., 2021).

Many studies have analyzed the diverse personal and situational factors impacting the vulnerability and resilience of persons amidst COVID-19 confinement measures (e.g., Amerio et al., 2020; Blustein and Guarino, 2020; Daly et al., 2020; Kuhn et al., 2021; Prati, 2020; Serafini et al., 2020; Xiong et al., 2020; Dale et al., 2021; Passavanti et al., 2021), but the relationships between leisure activities and housing conditions have thus far received little attention. However, it seems highly important to examine linkages between housing conditions, leisure activities, and mental health and well-being during the COVID-19 crisis.

Herein, the first Swiss lockdown is presented as a particularly interesting case because the country only imposed minor restrictions on activities outside the home. Relying on data collected during a national survey conducted as part of a broader study (for details, see Fritz et al., submitted), we examined the diverse impacts of lockdown measures and the potential role of social, economic and residential inequalities related to gender, age and employment status in exacerbating the deterioration of mental well-being during the COVID-19 crisis.

### The First COVID-19 Lockdown in Switzerland

At the beginning of the COVID-19 pandemic, the Swiss government implemented a set of measures to contain the virus and protect vulnerable persons Schweizerischer Bundesrat, 2020a,b). All public facilities deemed incapable of accommodating physical distancing measures were closed, including educational institutions, stores, restaurants, bars, concert halls, museums, libraries, cinemas, concert halls and theaters, sports centers, swimming pools, ski resorts, and hairdressing or beauty salons. Exceptions were only made for essential services such as grocery stores, health care facilities such as pharmacies, post offices, telecommunications providers, banks, gasoline service stations and workshops, and public transportation facilities. Employers were asked to take hygienic measures and maintain physical distance between workers as well as encouraged to allow personnel to work from home, and the state granted substantial financial means for short-term working compensation and emergency economic aid to sustain the economy and ensure that employees continued to receive wages. It was generally recommended to avoid public transport and maintain distance from others in public spaces. Persons older than 65 and those suffering from high blood pressure, diabetes, cancer, cardiovascular diseases, chronic respiratory diseases, and diseases that weaken the immune system were considered high-risk and encouraged to remain at home and avoid crowds and public transport (Swiss Federal Office of Public Health (SFOPH), 2020a). The Federal Council also called upon the people to stay at home, unless they had to go to work or were engaged in other essential activities, especially if they were old, belonged to a risk group or felt ill. These recommendations were promoted by slogans such as “*The Federal Council and Switzerland are counting on you!”* in order to *Help save lives* (SRF (Schweizerischer Rundfunk), 2020; Swiss Federal Office of Public Health (SFOPH), 2020b). However, unlike in neighboring Italy and France, there was no obligation or curfew requiring people to stay indoors. Rather than enforcing laws or imposing fines to dissuade people from meeting with groups exceeding five, the Swiss crisis response heavily relied on recommendations communicating a moral imperative to reduce social contacts and remain home. Thus, compared to other possible enactments, the first Swiss lockdown could be called a “semi-confinement.” However, evidence suggests that any lockdown measure has substantial negative effects on mental well-being (Brodeur et al., 2021). Therefore, it seems particularly interesting to examine who experienced emotional difficulties and how the living environment contributed to such conditions.

### Factors Influencing the Impact of COVID-19 Measures on Mental Well-Being

Extensive research has examined the influence of various aspects of employment status, housing conditions, leisure activities and sociodemographic features such as age and gender on the emotional state of persons during the COVID-19 pandemic. Previous studies elsewhere indicate that women, as well as young and single individuals particularly suffered during the crisis (e.g., Daly et al., 2020; Kowal et al., 2020; Li et al., 2020; Liu et al., 2020; Ranta et al., 2020; Dale et al., 2021; Passavanti et al., 2021), and a Swiss study by Kuhn et al. (2021) was consistent with these findings. People in partnered relationships may suffer less than singles from social isolation and confinement measures because they can receive positive social support from their partner, and have at least one close social contact whom they may frequently meet in-person (Burhamah et al., 2020; Jiang et al., 2021). Couples may also be in a more financially stable situation, as one partner may support another in case of income loss.

A glimpse into the impacts of the COVID-19 pandemic on the mental well-being of young adults was provided by Ranta et al. (2020), who reported that younger persons experienced greater anxiety regarding negative effects on their career, education and economic situation compared to older people, whose careers and financial situation tend to be more consolidated. A further reason may be that children and youth are still developing their identities, and they are generally more interested, open to and eventually dependent on diverse social contacts. A recent German study by Ravens-Sieberer et al. (2021) found extremely negative psychological impacts on a sample of children and adolescents, two-thirds of whom reported to be heavily burdened by the COVID-19 crisis, and they experienced a substantially higher prevalence of mental health problems and higher anxiety levels during the COVID-19 crisis compared with the time before the pandemic.

Researchers and media around the world have highlighted the additional constraints that women faced during the pandemic. Lockdowns exacerbated structural inequalities in domestic labor (Aldossari and Chaudhry, 2020; Shafer et al., 2020), caregiving (Power, 2020), and wages (e.g., Kristal and Yaish, 2020) as well as violence within the domestic sphere (Roesch et al., 2020). Pregnant women and women working in the health sector were particularly affected (Preis et al., 2020; Ceri and Cicek, 2021). In this regard, previous findings showing that people in partnership suffered less from the crisis need to be more nuanced. Although financial and social life may be easier for couples, women in heterosexual unions may have borne additional labor and stress.

People who lost their jobs due to the crisis suffered particularly strong negative impacts on mental well-being (Blustein and Guarino, 2020; Burhamah et al., 2020; Garre-Olmo et al., 2021). Kuhn et al. (2021) found that people experiencing the financial consequences of unemployment evinced the highest decrease in life-satisfaction among others in the sample. Loss of income and employment can engender social as well as financial insecurity, both of which have the potential to severely deteriorate an individual’s mental state (Hensher, 2020).

Housing conditions are another factor that could potentially moderate the psychological strain caused by the COVID-19 pandemic (Passavanti et al., 2021). Distancing measures and the closure of schools and workplaces as well as of locations for sports and leisure activities contribute to physical and social isolation, which has important implications concerning the role of housing conditions in supporting health and well-being (Tokazhanov et al., 2020). Studies on the relationship between housing characteristics and mental strain during the COVID-19 crisis have found that large housing space, high quality and comfort of the indoor area, access to an agreeable balcony, and having a green view were positively related to mental well-being (Amerio et al., 2020; Passavanti et al., 2021). Furthermore, the activities that people perform during the COVID-19 pandemic may also influence the psychological strain they experience. For example, spending great deal of time watching TV and following threatening media coverage of the pandemic focusing on aspects such as increasing numbers of cases, suffering of persons in intensive care, and reports on deaths may increase anxiety levels (Salari et al., 2020). Burhamah et al. (2020) accordingly found that intense consumption of COVID-19-related news was associated with higher levels of depression, and that an increased social media use during the COVID-19 crisis was related to higher levels of depression and anxiety. A review by Xiong et al. (2020) accordingly showed excessive exposure to news regarding COVID-19 and frequent use of social media to be associated with higher levels of psychological strain.

The positive effects of participation in sports and other forms of physical activity on health and well-being have long been established (Martin et al., 2009; WHO, 2010; Piercy et al., 2018; Anderson and Durstine, 2019; Woods et al., 2020). Regular exercise is an effective salutogenic means to reduce the risk of obesity and severe physical illnesses including diabetes, coronary heart disease, and some forms of cancer (WHO, 2010; Lachowycz and Jones, 2011). Many studies have also demonstrated that physical activity increases personal well-being and can reduce negative thoughts and depression symptoms (Avison and Turner, 1988; Biddle et al., 2000; Wunsch et al., 2017; Abdin et al., 2018), and several articles have reported on the beneficial role of physical activity in reducing depressive symptoms and other aspects of mental strain during the COVID-19 crisis (Garre-Olmo et al., 2021; Peterson et al., 2021). For example, Dale et al. (2021) found that individuals performing 30 min of physical activity at least once per week reported better mental health during the Austrian COVID-19 lockdown in December 2020 compared with those who did not exercise. Maugeri et al. (2020) similarly found a significant positive relation between individuals’ levels of physical activity and their well-being during a COVID-19 lockdown phase in Italy. A Canadian study by Lesser and Nienhuis (2020) found a positive impact of increased physical activity levels during the COVID-19 crisis on mental well-being among previously relatively inactive persons. Furthermore, the relevance of these findings is highlighted by various studies, which showed that the average level of physical activity significantly decreased in connection with the pandemic and confinement measures (e.g., Maugeri et al., 2020; Rhodes et al., 2020; Woodruff et al., 2021).

### Hypotheses of the Study

This study examined the changes in subjective psychological strain during the first lockdown in Switzerland. Following a review of the existing literature, several hypotheses were made in relation to moderating aspects.

An impressive amount of previous research supports our corresponding Hypothesis 1:

*Study participants will report a significant increase of psychological strain and decrease in well-being during the COVID-19 lockdown compared to before the crisis.*

This study strongly focused on the role of housing conditions and diverse types of activities in impacting mental well-being during the lockdown. It was supposed that people need adequate material conditions and positive mental dispositions in order to perform psychologically healthy activities. Therefore, by identifying such activities along with domestic and career inequalities, this research can contribute to developing recommendations or even campaigns, and supportive measures (e.g., Abdin et al., 2018; Lesser and Nienhuis, 2020). We examined diverse activities such as time spent watching television, playing, computer games, using social media, cooking and reading in an explorative way. However, based on the extensive body of research on physical activities presented in the previous section, we formulated the corresponding Hypothesis 2:

*Increased levels of physical activity are connected to lower levels of subjectively perceived psychological strain.*

Furthermore, we examined the moderating effect of the housing situation on the mental health impacts of the COVID-19 crisis. Home-office arrangements and recommendations urging people to spend more time at home may be more endurable if the surroundings are agreeable. Based on previous research findings (e.g., Amerio et al., 2020; Passavanti et al., 2021), we formulated Hypothesis 3:

*A lack of comfort in housing is connected to higher levels of subjectively perceived psychological strain.*

The employment status of the participants was also addressed in this study. In line with previous research (Blustein and Guarino, 2020; Burhamah et al., 2020; Kuhn et al., 2021; Garre-Olmo et al., 2021), it was expected that persons who lost their work or were temporally unemployed due to the lockdown would suffer the most. Loss of income and occupation can be connected to substantial declines in social status, financial wealth and self-esteem, which in turn may trigger severe psychological strain. Various further influences of the professional situation on mental strain seem possible (e.g., different levels of well-being among those working in home-office compared to those working in a presence mode). Therefore, we formulated Hypothesis 4 to state:

*Employment status during the crisis is significantly related to the level of subjectively perceived psychological strain.*

Furthermore, the lockdown measures and recommendations to stay at home led to increased social isolation. It seems plausible that singles suffer more strongly from social isolation compared to married or unmarried couples (Burhamah et al., 2020; Kuhn et al., 2021). Accordingly we formulated Hypothesis 5 as:

*The partnership status of a person is significantly related to the level of subjectively perceived psychological strain experienced during lockdown periods.*

Finally, based on a number of previous studies that have identified particularly severe negative psychological impacts among females and young adults (e.g., Daly et al., 2020; Kuhn et al., 2021; Ranta et al., 2020; Dale et al., 2021), we formulated hypotheses in relation to gender and age:

Hypothesis 6: *Females experienced higher levels of subjectively perceived psychological strain than males during lockdown periods.*

Hypothesis 7: *Young adults experienced higher levels of subjectively perceived psychological strain than older persons during lockdown periods.*

In addition to testing these hypotheses, the study also explored relationships between subjective psychological strain and further variables such as selected language of the questionnaire, education levels and certain aspects of employment status before the crisis.

## Materials and Methods

This study used data collected as part of a transformative, mixed methods study (cf. Creswell, 2009) that examined the material and emotional dimensions of individuals’ reactions to the COVID-19 crisis from a socio-ecological perspective with the aim of providing crisis support by giving access to targeted websites and hotlines and offering spaces for exchange and mutual learning. The École Polytechnique Fédérale de Lausanne (EPFL), University of Lausanne (UNIL), and the Idiap research institute were involved in the project. The overall research project comprised various components: a national online survey, semi-structured interviews, a mobile crowdsourcing application (app), and an interactive citizen science activity (see Fritz et al., submitted, for a detailed description of the mixed methods design). The project also offered services aimed at supporting individuals who had been negatively affected by the crisis.

### Questionnaire

The questionnaire used in this study asked for socio-demographic information, social, employment and housing aspects, and changes in the frequency of diverse activities and contained items addressing subjective psychological strains and well-being. Although the survey was not primarily designed from a mental health perspective, it assessed respondents’ emotional states, such as whether they were feeling “depressed,” “anxious,” “happy,” or “calm,” and asked for a variety of information on respondents’ domestic and daily lives. As such, it offers material to discuss and complement the existing literature on the psychological effects of the crisis. Moreover, the survey provided respondents with resources such as telephone numbers for psychological consultancy, reporting domestic violence, online sports classes or neighborhood solidarity networks.

The questionnaire started with questions on gender, age, highest completed education, partnership status, and employment status immediately prior to the crisis and at the time of the survey. Thereafter, questions on the housing situation asked participants about the number of individuals living in their household, the number of children and teenagers among these persons, and the number of rooms of the accommodation. Participants were also asked whether they lived in an urban or rural are (1 = *urban* to 4 = *rural*), whether their housing was lacking comfort (ranging from 1 = *no lack of comfort* to 5 = *substantial lack of comfort*), and whether their housing offered access to an outdoor space (e.g., balcony, terrace, private garden, shared garden).

Four consecutive items addressed changes in subjective psychological strain and well-being compared with before the crisis. Participants were asked whether they felt depressed (anxious, happy, calm) less often, equally often or more often at the time of the survey than prior to the COVID-19 lockdown. To align the direction of the rating of these four variables, the two items representing negative psychological states (depressed, anxious) were coded with the values −1 (*less often*), 0 (*equally often*), and 1 (*more often*) and the two positive items (happy, calm) were coded with the opposite poling. Finally, participants were asked whether they spent more or less time on six leisure activities: participating in physical activity, watching TV, playing video games, reading, using social media and cooking (four response options: *less time than before*, *more time than before*, *equally much time*, *not applicable*).

The survey was offered in three of the official languages of Switzerland (German, French, Italian) as well as English, and participants could select their preferred option.

### Data Collection

The survey was disseminated via a press release from the coordinating university EPFL to mainstream media in Switzerland, the university’s website, social media (Twitter and Facebook), and networks of the involved institutions. The goal of this proceeding was to reach and recruit many persons in order to gain a substantial data basis. The survey was administered from 8 April–10 May 2020; it began approximately 3 weeks after broad lockdown measures were introduced (16 March) and ended the day before most of the measures were terminated on 11 May. A total of 6909 persons visited the survey webpage and participated voluntarily in the survey after reading the information of the purpose of the study. No payment was offered or made for participation. The survey was quite long, and 977 (14.1%) participants dropped out prior to the last item included in this article. The final sample consisted of 5932 participants who completed the questionnaire items covered by this study, which encompass items ending briefly before the end of the overall questionnaire. However, the number of responses for certain items may be lower than others, as some items offered options such as “I do not want to answer this question” or “not applicable.”

### Statistical Analysis

The statistical analysis was done using IBM SPSS Version 26. Inferential statistical methods included chi-square tests to compare frequencies and a one sample *t*-test for the analysis of the significance of the deviation of a mean scale value from zero. Cronbach’s alpha reliability analysis was conducted in relation to the four-item scale developed to measure subjectively perceived increases of psychological strain. The average value of each participant’s responses over the four items was calculated as a measure for self-reported change in psychological strain ranging from −1 (decrease of psychological strain) to 1 (increase of psychological strain). To avoid an accumulation of missing values when calculating scale values, individual missing responses of participants were estimated by overall means of the corresponding item, if at least one of the four items was answered. Hypotheses were tested using an analysis of covariance (ANCOVA) general linear regression model that combined grouping variables and covariates for the prediction of the subjective psychological strain scale. The number of imputed missing values for the four items was low, ranging from 1.7% for feeling calm via 3.2% (happy), and 6.7% (anxious) to 9.4% (feeling depressed). However, omitting cases with missing responses in one of the scale items was not an option since this could have biased the findings systematically (c.f. Horton and Kleinman, 2007), because persons suffering from strong psychological strain may eventually have problems to concentrate and may thus have failed to respond to the survey completely. Imputation by mean values was selected since this method does not change average values of variables and tends to be neutral in relation to significance testing. Nevertheless, an additional sensitivity test of the significant determinants resulting from the ANCOVA model was calculated subsequently using only those persons who responded to all four items (*n* = 5347) in order to rule out the possibility of bias due to the imputation of missing values. Since only significant predictors emerging form the analysis of all cases were included, this sensitivity test additionally checked for stability of the findings when removing non-significant (i.e., irrelevant) components of the statistical model.

### Participants

Table 1 presents the sociodemographic details of the participants. The language distribution of the 5932 participants was 90% French, 5.1% German, 2.8% English, and 2.1% Italian. The survey was accordingly not representative of the Swiss population, but rather has a focus on the French speaking parts of Switzerland. The latter can be attributed to the recruitment paths of EPFL, UNIL, and Idiap, all of which are located in the French speaking part of the country and are hence more visible and better connected to media in this region.

**TABLE 1:** Sociodemographic details of participants.

Categories Variables	N	Percentage
**Selected language**		
French	5339	90.0
German	300	5.1
Italian	125	2.1
English	168	2.8
Total	5932	
**Gender**		
Male	2054	34.6
Female	3825	64.5
Other	10	0.2
No response	43	0.7
Total	5932	
**Age**		
18–24	589	9.9
25–34	1386	23.4
35–44	1514	25.5
45–54	1201	20.2
55–64	771	13.0
65–74	356	6.0
75 or more	86	1.4
No response	29	0.5
Total	5932	
**Education level**		
Low (compulsory education)	232	3.9
Medium (above compulsory, no academic degree)^*a*^	2492	42.0
High (university or applied university degree)	3208	54.1
Total	5932	
**Partnership status**		
Other (single, diverse partnerships, no response)	2055	34.6
Non-married couple	1459	24.6
Married couple	2418	40.8
Total	5932	
**Employment status immediately prior lockdown**		
Employed	4350	73.3
Student	480	8.1
Unemployed or retired	877	14.8
Other, no response	225	3.8
Total	5932	
**Employment status at the time of the survey**		
Job loss or temporary unemployment due to COVID-19	174	2.9
Unemployed	639	10.8
Home-office, distance working	2566	43.3
Hybrid home-office or distance working	771	13.0
Work on site	745	12.6
Other situation (e.g., retired), no response	1037	17.5
*N*	5932	

*^*a*^ Missing responses for education level were assigned to the medium category.*

The gender distribution of the sample was similarly unrepresentative for the Swiss population, as 64.5% of the participants were female. The participants were all 18 years or older, and the median and mode of the age distribution were between 35–44 years. More than half of the participants (54.1%) had earned an academic degree from a university or university of applied science, 42% had completed non-academic school or vocational formation or training beyond compulsory school, whereas 3.9% had only attended and/or completed compulsory school. Nearly 41% of the participants were married, 24.6% were in a relationship, and 34.6% were single. Approximately 73% of the participants were working immediately before the lockdown and 8.1% were students. Among participants who were employed, 2.9% had lost their jobs or were temporarily unemployed due to COVID-19, 43.3% worked in home offices or in other forms of distance telework, 13% were working in hybrid mode and 12.6% were working in-person with direct face-to-face contact with co-workers and clients.

Distributions of variables related to participants’ housing situation are shown in Table 2. Approximately 14% of the respondents reported living alone, 25% with one other person and 58% in households comprising three or more people. The majority of participants (57.3%) did not live with children or teenagers, and only about 10% lived in dwellings with less than three rooms. Participants mostly resided in rural areas and small villages (37.2%) rather than large cities (22.1%). Only 6.1% agreed or somewhat agreed that their housing lacked comfort; most had a private garden (23.8%), a private terrace or balcony (43.1%) or several exteriors (20.9%).

**TABLE 2:** Distribution of the responses of the participants in items referring to the housing situation.

Categories Item	N	Percentage
**Number of persons living in the household**		
Only me	851	14.3
2 persons	1495	25.2
3 persons	1264	21.3
4 persons	1103	18.6
5 persons or more	1065	18.0
Number fluctuates or no response	154	2.6
**Individuals under 20 in the household**		
0	3397	57.3
1	958	16.1
2	1103	18.6
3	254	4.3
4 or more	60	1.0
No response	160	2.7
**Number of rooms**		
1–1.5	134	2.3
2–2.5	476	8.0
3–3.5	1099	18.5
4–4.5	1501	25.3
5–5.5	1303	22.0
6 or more rooms	1393	23.5
No response	26	0.4
**Community type**		
Big city, urban area	1311	22.1
Urban periphery, suburban area	910	15.3
Small town, large village	1486	25.1
Countryside, rural area	2207	37.2
No response	18	0.3
**Do you have an outdoor space?**		
None	469	7.9
Other	39	0.7
Shared garden	204	3.4
Private garden	1413	23.8
Several exteriors	1239	20.9
Private terrace or balcony	2556	43.1
No response	12	0.2
**Rating-scale: “My housing lacks comfort”**		
Strongly disagree	4037	68.1
Somewhat disagree	602	10.1
In between	641	10.8
Somewhat agree	173	2.9
Strongly agree	189	3.2
No response	290	4.9

## Results

### Descriptive Statistics and Bivariate Relations

#### Variables Measuring Subjective Psychological Strain and Well-Being

A Cronbach’s alpha analysis of the four items concerning depressed, anxious, happy, and calm feelings revealed a reliability of α = 0.80, which indicates acceptable internal consistency. Omitting any of the four items from the scale would have decreased the Cronbach-α to values ranging from 0.73 to 0.77.

**FIGURE 1:**
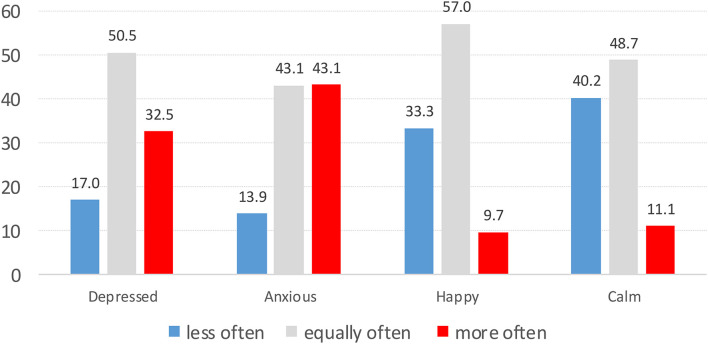
Percentage of participants experiencing feelings indicative of subjective psychological strain (depressed, anxious) and well-being (happiness, calm) less often or more often at the time of the survey than before the COVID-19 lockdown (n_*Depressed*_ = 5420, n_*Anxious*_ = 5558, n_*Happiness*_ = 5750, and n_*Calm*_ = 5835).

A considerable number of respondents reported feeling depressed (32.5%) and anxious (43.1%) more often at the time of the survey than before the COVID-19 lockdown. The response “more often” was significantly (both Chi-square tests, *p* < 0.001) more frequent than the complementary response “less often” for these two items capturing negative psychological change (depressed 17%, anxious 13.9%; Figure 1). For the two items capturing aspects of positive psychological change, the response “less often” occurred significantly more frequently (happy 33.3%, calm 40.2%) than the complementary responses “more often” (happy 9.7%, calm 11.1%; *p* < 0.001 for both chi-square tests).

Scale values for participants’ responses ranged from −1 to +1 with an average value of M = 0.24 (SD = 0.51; *N* = 5932) reflecting an increase in subjective psychological strain. The average value was significantly above zero (one sample *t*-test, *p* < 0.001). Thus, Hypothesis 1 was confirmed by responses to the overall scale measuring psychological strain as well as the four variables contained in the scale.

#### Variables Measuring Activity Changes

Table 3 presents the distribution of participants’ responses to items measuring changes in the frequency of performing certain activities. Whereas participation in physical activity significantly decreased during the lockdown, time spent watching TV, playing video games, reading, using social media and cooking increased. Chi-square tests comparing “less time” and “more time” responses for each activity were all significant (for all six tests, df = 1, *p* < 0.001). Thus, although the government did not impose stringent legal restrictions on activities outside the home, conditions did not necessarily offer the opportunity to adapt habits and invest substantial time in participating in sport activities.

**TABLE 3:** Percentage distribution and chi-square tests for increases (decreases) in the frequency of different types of activity.

Frequency change	Physical activity	Watching TV	Video Games	Reading	Social media	Time spent cooking
More time	25.8	54.1	20.8	43.1	58.2	63.9
Less time	45.1***	6.6***	2.3***	9.0***	3.2***	2.8***
Unchanged	25.8	34.5	16.6	38.4	30.0	30.9
Not applicable	3.3	4.8	60.3	9.5	8.5	2.4

***p < 0.001, two-sided chi-square tests comparing the observed number of cases reporting increases and decreases of each activity.

### ANCOVA Model Predicting Subjective Psychological Strain

A linear regression ANCOVA model was built to explain changes of subjective psychological strain during the COVID-19 crisis (Table 4). The model contained the grouping variables *language*, *gender*, *age group*, *education level*, *partnership status*, *employment status prior to lockdown*, *employment status at time of the survey*, *type(s) of outside space* of the housing, changes in the activities of *sport participation*, *watching TV*, *playing video games*, *reading*, *social media use*, and *cooking*. Covariate variables were *number of persons living in household*, *number of children or teenagers living in the household*, *number of rooms of the dwelling*, *number of rooms per person*, and the ratings for *location/community type* (from urban to rural), and *lacking comfort in housing*.

**TABLE 4:** ANCOVA model explaining subjective psychological strain.

			df	MS	F	Sign. p	Part. eta^2^
**Grouping variables**							
Language			3	0.35	1.54	0.202	0.00
**Gender**			**2**	**2.61**	**11.33**	0.000***	**0.00**
Age group			7	0.20	0.85	0.547	0.00
Education level			2	0.01	0.04	0.959	0.00
**Partnership status**			**2**	**0.76**	**3.31**	0.037*	**0.00**
**Employment status before crisis**			**3**	**0.75**	**3.24**	0.021*	**0.00**
**Professional situation during crisis**			**5**	**1.49**	**6.48**	0.000***	**0.01**
**Outside space**			**6**	**0.53**	**2.31**	0.031*	**0.00**
**Physical activity**			**3**	**12.28**	**53.29**	0.000***	**0.03**
**Watching TV**			**3**	**3.41**	**14.81**	0.000***	**0.01**
**Playing computer games**			**3**	**0.87**	**3.77**	0.010**	**0.00**
**Reading**			**3**	**4.27**	**18.53**	0.000***	**0.01**
**Social media networks**			**3**	**5.19**	**22.50**	0.000***	**0.01**
**Time spent cooking**			**3**	**1.35**	**5.87**	0.001***	**0.00**
Covariate variables	b	SE(b)					
Persons living in the household	−0,01	0,012	1	0.05	0.23	0.633	0.00
Children/teenagers in the household	0,00	0,010	1	0.03	0.12	0.731	0.00
Number of rooms	0,01	0,005	1	0.80	3.46	0.063	0.00
Number of rooms per person	0,00	0,008	1	0.02	0.10	0.748	0.00
Location (urban to rural)	0,00	0,006	1	0.01	0.05	0.819	0.00
**Lack of comfort**	**0,07**	**0,007**	**1**	**22.25**	**96.57**	0.000***	**0.02**
Error			5877	0.23			

*Significant predictor variables are in bold font.*

Gender, partnership status, employment status before and during the crisis, outside housing space(s), housing comfort, and changes in the frequency of all six activities were found to be significantly related to changes in subjective psychological strain during the COVID-19 crisis. The most influential variable was the time spent on physical activity (*p* < 0.001, η^2^ = 0.03). The average subjective psychological strain was 0.34 among those who spent less time on physical activity during the crisis than prior to it versus 0.11 among those who had increased their physical activity. Thus, Hypothesis 2 was confirmed.

The influence of frequency changes in the other five activities was less strong but also significant (Tables 4, 5). Both watching more and less TV than before the lockdown were connected to stronger increases of subjective psychological strain compared to no change in TV-watching frequency (Table 5). In addition, spending more time playing computer games and using social media were connected with increased psychological strain. In contrast, more time spent reading and cooking corresponded with less change in subjective psychological strain versus less time engaged in those activities.

**TABLE 5:** Mean values of the experienced changes in subjective psychological strain among persons with increases, decreases and no changes in the frequency of the performance of different types of activities.

	Physical activity		Watching TV		Video Games		Reading		Social media		Time spent cooking	
	M	SD	M	SD	M	SD	M	SD	M	SD	M	SD
More time	0.11	0.53	0.30	0.51	0.29	0.52	0.21	0.52	0.31	0.51	0.24	0.52
Less time	0.34	0.48	0.25	0.56	0.18	0.59	0.43	0.51	0.20	0.59	0.49	0.47
Unchanged	0.20	0.48	0.16	0.48	0.18	0.48	0.23	0.48	0.14	0.48	0.24	0.48
Not applicable	0.31	0.51	0.19	0.50	0.25	0.50	0.30	0.52	0.18	0.46	0.18	0.49

The second largest effect was found for the rating of lacking housing comfort (*p* < 0.001; *b* = 0.07, η^2^ = 0.02). Those who subjectively perceived a lack of comfort evinced an average subjective psychological strain of 0.48, whereas the average psychological strain among those who were fully satisfied with the comfort of their housing was only 0.20 (Table 6). Thus, Hypothesis 3 was supported.

**TABLE 6:** Mean values of changes in subjective psychological strain according to predictor variables.

Categories Predictor variables	*N*	M	SD
**Gender**			
Male	2054	0.19	0.47
Female	3825	0.27	0.52
Other/No response	53	0.28	0.47
**Partnership status**			
Other (single, diverse partnerships, no response)	2055	0.26	0.52
Unmarried couples	1459	0.22	0.52
Married couples	2418	0.24	0.49
Total			
**Employment status shortly before the lockdown**			
Employed	4350	0.23	0.51
Student	480	0.30	0.53
Unemployed or retired	877	0.25	0.47
Other, no response	225	0.35	0.46
Total			
**Employment status at the time of the survey**			
Job loss or temporary unemployment due to Covid19	174	0.37	0.54
Unemployed	639	0.25	0.49
Home-office, distance working	2566	0.21	0.53
Partial home-office, distance working	771	0.25	0.50
Work on site, presence work	745	0.29	0.47
Other situation (e.g., retired), no response	1037	0.27	0.48
**Do you have an outdoor space?**			
None	469	0.24	0.52
Other	39	0.33	0.54
Shared garden	204	0.26	0.54
Private garden	1413	0.21	0.49
Several exteriors	1239	0.21	0.51
Private terrace or balcony	2556	0.28	0.51
No response	12	0.42	0.40
**Covariate rating-scale: “My housing lacks comfort”**			
Strongly disagree (1)	4037	0.20	0.50
No response (Missing value estimate = 1.56)	290	0.27	0.47
Somewhat disagree (2)	602	0.28	0.50
In between (3)	641	0.36	0.51
Somewhat agree (4)	173	0.41	0.49
Strongly agree (5)	189	0.48	0.48

The availability and type of outside space attached to the dwelling was found to affect changes in subjective psychological strain (*p* < 0.05). Participants with multiple exteriors and those with access to a private garden adjacent to their homes experienced smaller increases in psychological strain. However, the housing location and the number of persons living in the household were not significantly related to psychological strain.

Current employment status exerted a significant influence on subjective psychological strain (*p* < 0.001), thereby confirming Hypothesis 4. Those who lost work due to the crisis evinced the highest increase in psychological strain, followed by those working in-person. The lowest increase was observed among persons working in a distance mode such as a home-office. Moderate increases were experienced by those working in hybrid conditions and those who were not working at the time of the survey for reasons unrelated to the crisis. Employment status immediately prior to the lockdown was significantly related to psychological strain (*p* < 0.05). The greatest increase in subjective psychological strain was observed among students, whereas less change was observed among people who had worked before the lockdown and those who were unemployed or retired.

Partnership status proved to be a significant factor (*p* < 0.05); singles reported the greatest increase in subjective psychological strain, followed by married couples. Thus, Hypothesis 5 was confirmed. Gender was also significantly related to increased subjective psychological strain (*p* < 0.001), which was lower among males. Thus, Hypothesis 6 was also confirmed.

Neither education level nor age were significantly related to reported changes in psychological strain. Hypothesis 7 assuming a particular strong subjective psychological strain among young participants was thus rejected. Nonetheless, it seems noteworthy that there emerged a non-significant tendency in line with the latter hypothesis as the youngest age group from 18–24 years (M = 0.28) was found to have the highest levels of subjective psychological strain, whereas the lowest levels were among those between 55–64 and those older than 75 years (M = 0.20 for both groups). Changes were moderate among the high-risk age category 65–74 years (M = 0.24).

### Sensitivity Analysis for Significant Predictors of Subjective Psychological Strain

To rule out the possibility of significant findings related to a possible bias due to the imputation of missing values, an additional linear regression ANCOVA model was calculated including only those cases (*n* = 5347) without any missing values in the four variables forming the subjective psychological strain scale. The significant predictors of the previous analysis entered the model as independent variables. The results confirmed the results of the previous ANCOVA model as all included variables proved to be significant predictors of subjective psychological strain in this supplementary ANCOVA test, which used a reduced sample and set of predictors. The variables gender, and professional situation during crisis, as well as physical activity, watching TV, reading, and social media network activities and cooking proved significant at the level of *p* < 0.001, partnership status and employment status before crisis at the level of *p* < 0.01, and outside space and playing computer games at the level of *p* < 0.05.

## Discussion

In this study, we operationalized emotional state and constructed an indicator to measure changes in subjective psychological strain and examined related factors among people in Switzerland during the first COVID-19 lockdown from mid-March to mid-May 2020. The findings show that depressive feelings and anxiety levels substantially increased during the confinement period, whereas indicators of mental well-being such as psychological calm and happiness substantially decreased. This finding is consistent with those of numerous other studies that identified negative effects of the COVID-19 pandemic and lockdown measures on mental well-being (e.g., Amerio et al., 2020; Daly et al., 2020; Serafini et al., 2020; Passavanti et al., 2021), thereby reinforcing the critical importance of considering the negative psychological effects of containment measures and providing supports to safeguard mental well-being and quality of life during similar situations in the future.

In addition to corroborating previous findings on factors moderating subjectively experienced psychological strain during the COVID-19 confinement, this study focused on often overlooked factors such as social and material domestic conditions as well as leisure activities. In the following sections, we will first discuss the findings on factors influencing the experienced psychological strain during the lockdown along with resulting implications for crisis management. Subsequently, we will address some general aspects of crisis management in terms of the processes leading to the development and definition of corresponding measures. Finally, we discuss the limitations of this study and future research needs.

### Factors Moderating the Effects of COVID 19 Crisis and Lockdown on Psychological Strain

In line with previous studies (e.g., Blustein and Guarino, 2020; Burhamah et al., 2020; Kuhn et al., 2021; Garre-Olmo et al., 2021), this study clearly showed that those who lost employment due to the pandemic have suffered particularly high levels of increased subjective psychological strain. In contrast those working full-time in a socially distanced mode such as a home-office reported the lowest increase in subjective psychological strain. Rather than a general plea for home-office, this result requires a nuanced interpretation; it might reflect the particularly difficult conditions experienced by those still working on-site, especially in the mostly feminine health and retail sectors, which echo the results on the impact of gender discussed below.

The strongest positive effect on maintaining subjective psychological well-being was exerted by an increase in physical activity during the lockdown. This finding is consistent with previous studies considering activities during the COVID 19 crisis as well as general findings on the positive effects of sports and other physical activities (Hansmann et al., 2007). Those reporting a behavior change of greater sport participation evinced the lowest increase of subjective psychological strain among all subgroups. Sedentary lifestyles characterized by low levels of physical activity and chronic stress have consistently been demonstrated to have a negative impact on health and well-being (WHO, 2010; Piercy et al., 2018; Anderson and Durstine, 2019). According to previous studies COVID-19-related social distancing and confinement measures have negatively impacted patterns of healthy activity (Rhodes et al., 2020; Woods et al., 2020; Woodruff et al., 2021). In line with this concern, a reduction of physical activities during the COVID-19 lockdown was observed among the participants of this study, and this change apparently had a negative effect on their well-being. In particular, physical activities in pleasant natural green spaces seem to be a promising means to increase individuals’ health and well-being and strengthen their immune systems (Frumkin, 2001, 2003; Martin et al., 2009; Duggal et al., 2018). Accordingly, policy-makers, urban designers, and landscape architects are encouraged to provide green-infrastructure, and forest and green space in urban and peri-urban areas to facilitate outdoor activities (Grahn and Stigsdotter, 2003; Belosi et al., 2021). However, there may be limits to the positive effects of physical activity. For example, Lesser and Nienhuis (2020) only found positive effects of increased physical activity during the pandemic among those who had previously not been very active.

Other activities were also related to levels of self-reported changes in psychological strain. Increased or stable frequencies of reading and cooking were associated with relatively low levels of increased psychological strain. Cooking may be a communicative activity that strengthens social relations in larger households as well as calming hobby that distracts attention from the mental burdens of the crisis. The latter consideration may also apply to reading.

However, we have to take into account that the directions of the causalities between activities and subjective psychological strain are not clear in this study. For example, studies have indicated that participating in sports and other forms of physical activity can help to reduce mental stress and depressive symptoms; however, at the same time, both stress and depression can prevent people from being physically active (Avison and Turner, 1988; Woodruff et al., 2021). Similarly, chronic stress, anxiety and depressive thoughts may also reduce the ability of persons to engage in hobbies such as reading and cooking, which require some level of mental calm. Nevertheless, the findings of this study suggest that reading and cooking may effectively mitigate the negative psychological effects of the COVID-19 crisis.

Activities involving the use of technology and media (watching TV, social media use, and computer games) were associated with similar patterns of findings; those who reported the same frequency of these activities as prior to the confinement experienced the lowest increase in subjective psychological strain, whereas those who began engaging in these activities more frequently experienced the greatest increase and those who spent less time had values in the middle. These findings point to a relationship between disturbed habits and stress. Individuals whose social and material conditions enabled them to maintain prior routines might have been more spared from substantial distress (Clément et al., (in press)). However, any recommendations made in relation to these activities may need to consider the content of the media and the degree of social embeddedness involved (cf. Burhamah et al., 2020; Salari et al., 2020).

We found that women reported greater increases in subjective psychological strain than men during the lockdown, which aligns with many previous studies (e.g., Daly et al., 2020; Li et al., 2020; Liu et al., 2020; Dale et al., 2021; Passavanti et al., 2021) that have indicated that the COVID-19 crisis reinforced previously existing gender inequalities. Although working from home appears to provide some relief from psychological strain, women may have taken on additional domestic labor related to childcare and education, especially if they previously relied on paid housekeepers whose ability to be present became complicated by sanitary restrictions. On the other side, because the professional sectors most exposed to the virus have largely been female-dominated, women still going to their workplaces would face cumulative factors of constraint related to domestic labor and professional stress. Therefore, gender inequalities should be carefully considered and actively countered when designing COVID-19 countermeasures and supportive interventions, especially with regard to childcare and homeschooling. This observation also highlights the need for public policies to tackle gender inequalities more generally. It should be noted that Switzerland has historically taken a conservative approach to women’s social roles through policies that reinforce a traditional gendered division of labor (Martin, 2020).

In contrast to previous studies (e.g., Ranta et al., 2020), we observed no significant effect of age on perceived increase of psychological strain during the crisis. However, similar to previous studies (e.g., Kuhn et al., 2021; Dale et al., 2021), the youngest age group was found to have the greatest increase in psychological strain. Furthermore, an implicit age effect was detected, as we identified a relationship between student status and increased psychological strain. This result is consistent with some previous studies and seems to be linked to findings demonstrating that social interaction is particularly important for young adults and that students may fear a negative impact of confinement on their (future) careers, which are still at an early stage (Ranta et al., 2020; Xiong et al., 2020).

Finally, a major finding of this study was the strong and highly significant relationship between housing comfort and the level of subjective psychological strain experienced during the crisis. Those who strongly agreed that their housing lacked comfort reported great increases in psychological strain. This result highlights an important social (in-)justice issue, as it implies that those who can afford comfortable housing have suffered less from the crisis. The type and availability of access to outdoor space attached to the housing was also significantly related to the level of increased psychological strain, as participants with a private garden and various exterior spaces (e.g., garden plus balcony) reported lower levels of increased psychological strain than those living in dwellings lacking these amenities. During a lockdown, people spend substantially more time in their homes, as activities usually taking place elsewhere, such as working, studying or relaxing, are transferred to domestic spaces. This finding adds to previously elucidated requirements that dwellings should meet to ensure people’s well-being (Tokazhanov et al., 2020). Corroborating previous studies (Amerio et al., 2020; Passavanti et al., 2021), our findings suggest that living in housing that is lacking in comfort and enjoyable outdoor space may exacerbate the mental burden of lockdown measures and complicate occupants’ lives during such times.

### General Implications for Crisis Management

It is important to carefully weigh prospective negative impacts on mental health against expected positive effects of containment measures. Doing so requires taking economic consequences of lockdowns into account, as unemployment and a deteriorating financial situation can trigger severe psychological problems (Fore, 2020; Hensher, 2020). Accordingly, along with politicians, epidemiologist and virologists, economic analysts, psychiatrists, psychologists, sociologists and other experts should be involved and consulted in the political discussion and decision-making processes when developing health protection measures to address pandemics. One way to ensure that multiple perspectives are equally considered could be to include professionals with diverse backgrounds and fields of experience and expertise in the processes leading to more socially robust decisions (Hansmann, 2010; Seidl et al., 2013). The interaction and mutual learning between experts, politicians, the public, and other stakeholders could contribute to a greater consideration of the diverse and unequal effects of confinement on ordinary people. The relationship between housing comfort, social situation, gender, and employment status, and psychological strain experienced during the lockdown reinforces the importance of social justice measures and achieving gender equality in times of crisis (Hensher, 2020; Kuhn et al., 2021; Mikolai et al., 2020). The Swiss government took immediate and clear actions to support the economy and compensate people in the event of income reduction and job losses; however, socially disadvantaged groups should also be involved in planning crisis measures. Similarly, the input of children and youth as well as very elderly persons seem to have been under-represented, and these groups have largely been excluded from the decision-making on COVID-19 mitigation. In-depth social scientific research and transdisciplinary approaches are needed to better understand the needs of younger people and the elderly and include them in the development and evaluation of protective measures.

### Limitations and Future Research Avenues

The declines in psychological well-being documented in this study occurred during the relatively brief period of the first Swiss COVID-19 lockdown. The negative consequences of containment measures may increase with the length of their duration (Garre-Olmo et al., 2021). Studies in Austria have shown that mental health problems were considerably more pronounced during the second lockdown from autumn 2020 to winter 2020/21 than during the first lockdown in spring 2020 (Dale et al., 2021). A comprehensive study on the impact of the second lockdown on mental well-being in Switzerland is needed.

Another limitation of this study is the exclusion of children and youth from the survey. Research on the negative impacts of confinement measures on children is urgently needed. Emerging studies in this regard suggest that children have suffered particularly strongly from COVID-19 countermeasures (Fore, 2020; Ravens-Sieberer et al., 2021), as confinement can make it difficult for them—as well as fragile elderly persons—to fulfill their basic need for social interaction (Maslow, 1954). Any efforts to design measures to contain future pandemics need to consider their situation and provide supports to ensure their psychological well-being.

## Data Availability Statement

The raw data supporting the conclusions of this article will be made available by the authors, without undue reservation.

## Ethics Statement

The studies involving human participants were reviewed and approved by Human Research Ethics Committee (HREC) of EPFL. The patients/participants provided their written informed consent to participate in this study.

## Author Contributions

LF, GC, AP, and CB developed and implemented the survey. RH was leading the statistical analyses and the drafting of the manuscript as well as further elaborations and revisions. All authors made substantial contributions to the conception and design of the work and interpretation of data and drafting of the work and revising it.

## Conflict of Interest

The authors declare that the research was conducted in the absence of any commercial or financial relationships that could be construed as a potential conflict of interest.

## Publisher’s Note

All claims expressed in this article are solely those of the authors and do not necessarily represent those of their affiliated organizations, or those of the publisher, the editors and the reviewers. Any product that may be evaluated in this article, or claim that may be made by its manufacturer, is not guaranteed or endorsed by the publisher.
